# Internalizing Symptoms in Developmental Dyslexia: A Comparison Between Primary and Secondary School

**DOI:** 10.3389/fpsyg.2020.00461

**Published:** 2020-03-24

**Authors:** Sara Giovagnoli, Luca Mandolesi, Sara Magri, Luigi Gualtieri, Daniela Fabbri, Eliana Tossani, Mariagrazia Benassi

**Affiliations:** ^1^Department of Psychology, University of Bologna, Bologna, Italy; ^2^Child Neuropsychiatry Unit, Azienda Unità Sanitaria Locale (AUSL) della Romagna, Forlì-Cesena, Italy

**Keywords:** dyslexia, internalizing symptoms, anxiety, depression, somatic complaints, psychological protection factors

## Abstract

Although the relationship between developmental dyslexia (DD) and the risk of occurrence of internalizing symptomatology has been widely investigated in the extant literature, different findings have been reported. In this study, two experiments with two general purposes are presented. The first study investigates whether the differences in the severity of internalizing symptoms between DD and controls are greater in students attending secondary school than in those attending primary school. Sixty-five DD and 169 controls attending primary and secondary school took part in the first study. The diagnosis of dyslexia was obtained from standardized reading tests; internalizing symptom severity was assessed with the Self Administrated Psychiatric Scales for Children and Adolescents questionnaire. The results showed that adolescents with dyslexia had an increased level of self-perceived anxiety, depression and somatic symptoms, whereas no significant differences between DD and controls emerged in childhood. In the second study, a cohort of adolescents attending secondary school (DD = 44; controls = 51) was closely analyzed to clarify whether contextual and subjective factors could contribute toward exacerbating the risk of internalizing symptomatology at that age. Internalizing symptom severity was assessed with the Child Behavior Checklist, Youth Self Report questionnaire, decision-making factors were measured with the Melbourne Decision Making Questionnaire, and student’s quality of life was gaged using the Clipper test. The results showed that high levels of internalizing symptoms in DD were associated with a low level of self-esteem and the tendency to react to problematic situations with hyperactivation. By contrast, positive relationships with peers were associated with low symptom severity. In conclusion, the intensified internalizing symptoms that could emerge in adolescents in association with the presence of dyslexia are predicted by social protective and risk factors that are associated with symptom severity. Accordingly, the results suggest that remediation programs for dyslexia should include implementing motivation strategies, self-esteem enhancement activities and building peers networks that, starting in childhood, can prevent the appearance of internalizing symptoms.

## Introduction

Developmental dyslexia (DD) is defined as a specific difficulty in fluency and accuracy of the grapheme-phoneme transcoding process (Lyon et al., 2003), despite normal intelligence, appropriate education and adequate socio-economic status. Out of all the learning disorders, DD is the most frequent (ICD-11, World Health Organization, 2018), affecting 3–17% of the school-age population (Vellutino et al., 2004).

A growing number of studies have confirmed the role of DD as a risk factor for high levels of internalizing symptomatology in children and adolescents (Biederman et al., 1991; Cantwell and Baker, 1991; Faraone et al., 1993; Beitchman and Young, 1997; Bäcker and Neuhäuser, 2002; see Mugnaini et al., 2009 for a review). However, some studies reported no significant differences in emotional symptomatology between DD and controls (Jorm et al., 1986; Lamm and Epstein, 1992; Newcomer et al., 1995; Miller et al., 2005; Maag and Reid, 2006).

From an empirically derived perspective of child behavior classification, anxiety, depression, social withdrawal and somatic complaints are conceptualized as internalizing problems (Achenbach and Edelbrock, 1978). With regard to anxiety symptoms, children with DD show a higher rate of separation anxiety and generalized and social anxiety compared to controls (Carroll et al., 2005; Goldston et al., 2007; Mammarella et al., 2014). High rates of school-related stress and anxiety were found in samples of secondary school children (Geisthardt and Munsch, 1996; Wenz-Gross and Siperstein, 1998) and high-school adolescents (Goldston et al., 2007). Specifically, in a meta-analysis of studies concerning school-age subjects with learning disabilities, In details, the association between DD and anxiety disorders is not due to familial influences or environmental risks (Willcutt and Pennington, 2000). Nelson and Harwood (2011) found that approximately 70% of children with DD reported high levels of anxiety symptoms. However, the same meta-analysis pointed out that there was high heterogeneity in terms of magnitude and direction of the effects reported in the included studies.

A similar pattern was found in relation to depressive symptomatology studies. Mammarella et al. (2014) investigated a sample of 8–16-years-old in a clinical setting, and Maughan et al. (2003) conducted a longitudinal study of boys aged 7 and 10 years. Both studies found robust links between severe, persistent reading problems and an increased risk of depressed mood. In contrast, Carroll et al. (2005) found that literacy difficulties were not significantly associated with diagnosable levels of depression in a sample of subjects aged 5 to 15 years old, but were associated with self-reports of depressed mood measured at 11–15-years old. In particular, the authors found significant gender and age effect: only 11-15-years-old male participants with literacy difficulties showed increased levels of depressed mood in a self-report questionnaire.

The association between learning disorders and somatic symptoms has been investigated in few studies (Willcutt and Pennington, 2000; Undheim, 2003; Arnold et al., 2005). These studies reported higher levels of somatic symptoms in DD participants compared to controls. More specifically, headache and stomach ache were frequently associated with DD. Willcutt and Pennington (2000) hypothesized that somatic symptoms could be considered a reaction to high levels of academic distress perceived by DD participants.

Some authors have suggested that the inconsistent results in the extant literature could be explained by the fact that risk factors were not sufficiently considered and controlled for. Although many studies have aimed to investigate the role of risk factors in deepening internalizing symptoms associated with DD, the majority were focused on clinical aspects. Mugnaini et al. (2009), reviewing previous studies, emphasized that the severity of dyslexia, the heterogeneity of symptoms, the comorbidity with other disorders (e.g. ADHD) and late diagnosis are major risk factors for the emotional suffering of children with dyslexia.

The lack of significant differences between DD and controls could also be attributed to the age of the samples included in the various studies. In line with this, a review conducted by Avenevoli et al. (2008) concerning community samples, indicated that the prevalence estimate of depression in children (approx. 7–12 years) is lower than in adolescents (approx. 13–18 years). Moreover, other authors found an increase in emotional symptomatology with increasing age in participants with learning disorders (Raskind et al., 1999; Wilson et al., 2009; Klassen et al., 2011).

The adolescence, coupled with contextual and environmental factors associated with the school learning environment, could be deemed to be a risk factor associated with the deepening of internalizing symptoms. Environmental changes are crucial in the transition from primary to secondary school since during this period the student goes from childhood to adolescence. In secondary school, teachers and rules are stricter, academic demands are higher, and social relationships change: relationships with adults become more formal and relationships with peers become more challenging. It is well known that different somatic and clinical manifestations have different ages of onset and progression (Angold et al., 2002; Silverman and Field, 2011; Kessler et al., 2012). The cause why internalization symptoms in DD are higher than usual during adolescence is still not fully understood (Angold et al., 2002; Silverman and Field, 2011). Surprisingly, only a few studies focused on the relationship between transitioning from primary to secondary school and warning signs of emotional suffering in children and adolescents with dyslexia. A longitudinal study conducted by Ackerman et al. (2007) on a sample of low economic status children found that teachers reported a specific association between reading problems and internalizing symptoms in 5th grade but not in 3rd grade students. This finding indicates that reading problems have an impact on the child’s emotional wellbeing only around the fifth grade, in the beginning of adolescence. Furthermore, the emotional distress associated with DD seems to continue in adulthood (Orth et al., 2008; Wilson et al., 2009; Klassen et al., 2011). A recent meta-analysis conducted on 15 studies examined the association between internalizing problems and learning disabilities in adulthood. The results showed a negligible change in the magnitude of internalizing problems in adults with DD, in comparison with children and adolescents (Klassen et al., 2011). Nevertheless, the authors suggested that the enduring nature of the learning disability could continue to influence individual psychological functioning after the end of formal schooling.

Although there is a generalized interest in DD policies and remediation programs in developed countries, it has been reported that emotional distress experienced by students with learning disorders frequently goes untreated (Bender and Wall, 1994; Sabornie, 1994; Rock et al., 1997; Sako, 2016). This lack of attention to emotional disturbances in learning disabilities, together with a lack of motivation and low self-esteem of individuals with reading difficulties, could constitute further risk factors for the development of internalizing symptoms during adolescence. It is thus crucial to understand whether different levels in self-reported psychological factors (i.e. self-esteem, perceived quality of life, social support and decision-making strategies), as well as general cognitive resources, could have an impact on internalizing symptoms in DD during adolescence. In the present study, we describe two experiments that investigated the main risk factors for the development of internalizing symptoms in dyslexia.

## Experiment 1

In the first experiment, we investigated whether the educational stage (primary versus secondary school) contributes to exacerbating the internalizing symptomatology in DD as compared to controls. The evaluation setting (clinical vs. school) was considered as a possible explanatory variable of the differences. We hypothesized that in young people with DD, the emotional changes that accompany the transition from primary to secondary school could constitute a contextual risk factor for mood disorders, anxiety or somatization. A detailed analysis of internalizing symptom severity was carried out using the SAFA questionnaire (Self Administrated Psychiatric Scales for Children and Adolescents questionnaire; Cianchetti and Sannio Fancello, 2001), which is a validated self-reporting scale that measures a wide range of anxiety, depression and somatic related symptoms by different subscales. Moreover, using normative data, it was possible to identify cases above the clinical cut-off point. It is interesting to note that several studies indicate that the levels of internalizing symptoms emerging from self-report questionnaires tend to be higher than those that emerge when parents have to identify the presence of emotional symptoms in their children (Bird et al., 1992; Stanger and Lewis, 1993; Epkins, 1996). It is therefore suggested that self-reporting measures could be used as an initial screening tool for the identification of emotional symptoms. For this reason, we based our analysis on self-reporting measures only.

### Methods

#### Participants

The sample was composed of 234 subjects (127 male, 207 female) aged between 8 and 16 years. The control group (C) was composed of 169 students (90 males and 79 females, mean age = 11.17, SD = 1.4), while 65 students belonged to the Developmental Dyslexia (DD) group (37 males and 28 females, mean age = 11.37, SD = 1.50). Eighty subjects attended primary school (8–11 years old; mean age = 9.64, SD = 0.93) and 154 attended secondary school (11-16 years old; mean age = 12.05, SD = 0.86) (see Table 1). Data were collected in part during a screening project for learning disabilities (*N* = 184) in three primary and two secondary schools located in Italy (Istituto Comprensivo di Mercato Saraceno, Istituto Comprensivo di Sarsina, Italy), and in part (*N* = 50) during the first day of diagnostic evaluation at the clinical service provided by the Child and Adolescence Neuropsychiatry Unit at Bufalini Hospital, Cesena, Italy. Forty-four of the DD students were evaluated after the school screening project (i.e. a screening evaluation for identifying subjects having reading disabilities), and 21 of the DD students were collected at the Neuropsychiatry Unit of the hospital. All the children in the sample were of Italian origin.

**TABLE 1:** Descriptive statistics of the sample of experiment 1.

Group	C (*n* = 169)		DD (*n* = 65)	
School (n)	Primary	Secondary	Primary	Secondary
Males	43	47	13	24
Females	18	61	6	22
**Setting (n)**				
School	43	97	9	35
Hospital	18	11	10	11
Age (Mean ± SD)	9.69(±0.940)	12.01(±0.86)	9.47(±0.91)	12.15(±0.87)
CPM Raven’s IQ	109.25(±13.5)	108.56(±11.01)	104.63(±10.16)	106.22(±10.68)
**Reading Tests (Mean ± SD)**				
W-R time	−0.23(±0.930)	0.11(±0.75)	2.50(±2.33)	1.77(±1.24)
W-R errors	0.02(±0.67)	0.10(±0.64)	1.80(±1.49)	2.32(±2.22)
NW-R time	−0.14(±0.96)	−0.20(±0.97)	1.33(±1.70)	1.35(±0.93)
NW-R errors	−0.16(±0.77)	−0.16(±0.92)	0.85(±1.19)	1.74(±1.90)

*Means and standard deviations (SD) of Age, General intelligence (CPM Raven’s IQ) and Reading tests parameters (Z scores) are reported. W-R time: time in word reading; W-R errors: errors in word reading; NW-R time: time in non-word reading; NW-R errors: errors in non-word reading.*

The inclusion and exclusion criteria adopted were those recommended by Consensus Conference on Specific Learning Disorders promoted by the Italian National Institute of Health (Lorusso et al., 2014) for diagnosis of developmental dyslexia. The inclusion criteria were based on standardized reading test (DDE-2; Sartori et al., 2007), specifically, accuracy and/or speed z-score below two standard deviations from the normative score in at least at one of the two reading tests (subtest 2 or 3, for the detailed description, see the “Instruments” section) were used as indicator of diagnosis of dyslexia. Table 1 reports the descriptive statistics of general intelligence and reading test scores for the controls and DD group. The exclusion criteria were: IQ cut-off lower than 70 presence of referred sensory disability, presence of attention deficit or hyperactivity disorder (ADHD) based on the DSM-5 recommendations (American Psychiatric Association, 2013). Informed consent was appropriately obtained from the parents of all participants.

#### Instruments

Reading level was assessed using subtests 2 and 3 from the Battery for the Evaluation of Developmental Dyslexia and Dysorthography (Sartori et al., 2007), an Italian standardized and validated test battery for the diagnosis of dyslexia. During the reading tasks, the subjects were required to read aloud, as quickly and accurately as possible, four lists of 28 words either with high or low frequency (4 to 8 letters long) and three lists of 16 non-words (5 to 9 letters long). The accuracy (number of errors) and speed (syllables/seconds) were evaluated for each subtest. Raw scores were converted into z-scores, according to standardized reference data; normative data are available separately for each grade from second to eighth grade.

The Colored version of the Raven Progressive Matrices was administered to evaluate the general intelligence of the children up to 11 years (CPM – Raven, 1994), while the Standard version of the Raven Progressive Matrices (SPM – Raven, 2008) was adopted for the students over 12. Raven’s CPM and SPM are common measures of basic cognitive functioning, quantifying a child’s ability to form perceptual relations and to reason by analogy, independent of verbal abilities and formal schooling. The CPM matrices comprise 36 items divided into three sets of 12 items (A, Ab and B), while SPM is composed of 60 items divided into five sets of 12 (A, B, C, D, E). The items are ordered by increasing difficulty. Each item is presented as a colored or black and white pattern with a missing portion, together with six or eight options for filling in the missing element. Some items test the ability to complete a continuing pattern, while others require the perception of the parts of the whole pattern as one gestalt on the basis of spatial relations. Finally, some items require analogical reasoning. Raw scores were converted to standard scores, according to Italian standard reference data.

The SAFA (Self Administrated Psychiatric Scales for Children and Adolescents; Cianchetti and Sannio Fancello, 2001), an Italian validated questionnaire, was used to assess clinical and subclinical internalizing symptomatology. SAFA is a self-reporting questionnaire for investigating the specific components of anxious, depressive and somatic symptoms. Many Italian studies have used these scales to assess different symptomatology associated with disorders like Tourette Syndrome, eating disorders or learning disabilities (Franzoni et al., 2009; Termine et al., 2011; Nacinovich et al., 2012; Pellicciari et al., 2012; Mammarella et al., 2014). The SAFA scales measure: Anxiety (SAFA-A), Depression (SAFA-D), Obsessive-Compulsive symptoms (SAFA-O), Psychogenic eating disorders (SAFA-P), Somatic Symptoms and Hypochondria (SAFA-S) and Phobias (SAFA-F). All of these scales were developed on the basis of DSM- IV criteria. In this study, we decided to use only the subscales for Anxiety (SAFA-A), Depression (SAFA-D) and Somatic symptoms/Hypochondria (SAFA-S), in line with previous findings in the literature. SAFA-A is composed of 42 items for children and 50 items for adolescents, and comprises the following subscales: Generalized Anxiety, meaning tension/uneasiness and apprehensiveness, preoccupation about the future; Social Anxiety, which investigates the characteristics of the avoidant disorder; Separation anxiety, which is related to separation in the literal sense, apprehension about loss and abandonment; and School-related Anxiety, which is specifically associated with worriedness about school life. SAFA-D is composed of 48 items for children and 56 for adolescents, and it comprises the following subscales: Depressed mood; Anhedonia and disinterest; Irritable Mood; sense of Inadequacy and low self-esteem; sense of Insecurity; sense of Guilt; and sense of Hopelessness. SAFA-S is composed of 20 items for children and 25 for adolescents, and comprises the following subscales: Somatic Symptoms, namely those related to the cardiac, gastrointestinal and respiratory systems, asthenia, sleep, general cenesthesis, and memory/concentration; and Hypochondria, which regards worriedness about illness. The subjects were asked to consider to what extent they agree with the content of each item, indicating whether the statement in question is “true,” “false” or “in between.” For SAFA-S, in which the items describe physical symptoms, the subjects had to indicate how often they experience those symptoms: “often,” “sometimes” or “never.” A scale is also provided to evaluate the subjects’ tendency for simulation. The SAFA questionnaire was validated on an Italian sample (895 children and adolescents aged 8 to 18 years), showing a high level of sensitivity, specifically for subclinical symptomatology. This aspect proved very useful for this study because, in the DD population, we expected to find subclinical symptomatology more frequently than clinical symptomatology. The raw scores of each scale and subscale were transformed into t-scores, using normative data. Whenever a t-score is equal to or higher than 70, it indicates a risk of clinical psychopathology (Cianchetti and Sannio Fancello, 2001).

#### Procedure

The students were individually assessed by an expert psychologist in two different sessions. In the first session, reading and cognitive performances were measured, while the second session evaluated internalizing symptoms (SAFA).

### Statistical Analysis

First, a multivariate analysis of variance (MANOVA) was performed on Anxiety, Depression and Somatic Symptoms global scales, considered as dependent variables, using the Group (DD vs. C), the School Level (primary vs. secondary), the type of setting (school vs. clinical setting) as between-subject factors. To describe the differences between groups in the SAFA subscales, three MANOVA’s were performed separately (one for Anxiety, the second for Depression, and the last for the Somatic Symptoms subscales); the subscale scores were used as dependent variables, while the Group (DD vs. C), the School Level (primary vs. secondary), and type of setting (school vs. clinical setting) were adopted as between-subject factors. The MANOVA assumptions (multivariate normality, equal covariance matrices across groups, and uncorrelated model errors) have been carefully checked and met. The results are described in Supplementary Material. Subsequently, we compared the frequency of clinically relevant scores (*t*-score equal to or higher than 70) in the DD and C groups in primary and secondary school using Fisher’s exact test.

### Results

The Multivariate Analysis of Variance showed that students with dyslexia had a higher level of anxiety [Group effect: *F*(1,226) = 5.67; *p* = 0.018; partial η2 = 0.024] and depression [*F*(1,226) = 5.14; *p* = 0.024; partial η2 = 0.022] as compared to controls (see Table 2), while no differences were found for somatic symptoms [*F*(1,226) = 1.69; p = 0.20; partial η2 = 0.01]. Depression and somatic symptoms were higher in secondary than in primary school [Depression: *F*(1,226) = 11.52; *p* = 0.001; partial η2 = 0.048; Somatic Symptoms: *F*(1,226) = 7.75; *p* = 0.006; partial η2 = 0.033]. Anxious symptoms were also higher in secondary school although not significantly [Anxiety: *F*(1,226) = 3.15; *p* = 0.077; partial η2 = 0.014]. As expected, we found higher scores in the clinical than in the school screening setting [Anxiety: *F*(1,226) = 10.54; *p* = 0.001; partial η2 = 0.045; Depression: *F*(1,226) = 6.45; *p* = 0.012; partial η2 = 0.028; Somatic Symptoms: *F*(1,226) = 4.82; *p* = 0.028; partial η2 = 0.021].

**TABLE 2:** Estimated marginal means (and standard errors_SE) of SAFA scales in Control (C) and Developmental Dyslexia (DD) group.

			School grade			Setting		
			Primary	Secondary		Screening	Clinical	
				
	Mean (SE)	*F*	Mean (SE)		*F*	Mean (SE)		*F*
**Anxiety**								
C	52.1(1.1)		52.7(1.4)	51.5(1.6)		48.3(0.9)	55.9(2.0)	
DD	56.5(1.5);	*F* = 5.7**	52.6(2.3)	60.3(1.8);	*F* = 6.0**	54.3(0.9)	58.7(2.3);	*F* = 0.75
Overall	54.3(0.9)		52.6(1.4)	55.9(1.2)	*F* = 3.1	51.3(1.1)	57.3(1.5);	*F* = 10.5**
**Depression**								
C	50.2(1.0)		48.1(1.3)	52.2(1.5)		46.2(0.9)	54.2(1.8)	
DD	54.0(1.4)	*F* = 5.1**	50.3(2.2)	57.7(1.6);	*F* = 0.9	53.7(1.8)	54.3(2.1);	*F* = 4.7**
Overall	52.1(0.8)		49.2(1.3)	55.0(1.1);	*F* = 11.5**	49.9(1.0)	54.2(1.4);	*F* = 6.4**
**Somatic**								
C	50.9(0.9)		49.7(1.2)	52.2(1.3)		47.5(0.8)	54.4(1.6)	
DD	52.9(1.2);	*F* = 1.7	50.0(1.9)	55.7(1.4);	*F* = 1.2	53.1(1.6)	52.6(1.8);	*F* = 6.3**
Overall	51.9(0.7)		49.8(1.1)	54.0(1.0);	*F* = 7.5**	50.3(0.9)	53.5(1.2);	*F* = 4.8**

*p < 0.05. **p < 0.01.

Furthermore, as described in Table 2, in the DD group, anxiety symptoms were higher in secondary school, while in the control group the symptomatology levels were lower [Group by School level interaction effect: *F*(1,226) = 5.98; *p* = 0.015; partial η2 = 0.026]. This interaction effect was not significant for depression [Group by School level interaction effect: *F*(1,226) = 0.88; *p* = 0.350; partial η2 = 0.004] nor for somatic symptoms [Group by School level interaction effect: *F*(1,226) = 1.21; *p* = 0.273; partial η2 = 0.005], which were higher in secondary school in a similarly way to the DD and controls.

Separate MANOVAs were performed for the anxiety, depression and somatic symptoms subscales, revealing that the DD group had a globally higher level of symptoms as compared to the controls for the majority of the subscales (see Supplementary Material for a detailed description of analyses on SAFA subscales).

We then estimated the frequency of cases with clinically relevant scores in each symptom scale when the subject’s *t*-score was above 70 (two standard deviations above the normative value). As depicted in Table 3, only the DD group attending secondary school exhibited a significantly higher percentage of cases with clinically relevant scores, as compared to controls, on anxiety (Fisher’s exact test; *p* < 0.01) and on depressive symptoms (Fisher’s exact test; *p* = 0.01). In primary school, no differences between groups were found on anxiety (Fisher’s exact test; *p* = 1.00). No subjects (both in C and in DD group) with relevant clinical scores of depression and somatic symptoms were found in primary school. Regarding somatic symptoms, the percentage of clinically relevant scores found in the two groups was not significantly different in secondary school (Fisher’s exact test; *p* = 1.0).

**TABLE 3:** Percentages of clinically relevant scores (t score above 70) of the SAFA scales in Control (C) and Developmental Dyslexia (DD) children.

SAFA scale% (n)	Primary school		Secondary school		Total	
	C	DD	C	DD	C	DD
Anxiety	4.9% (3)	5.3% (1)	**2.8%(3)**	**23.9% (11)**	**3.6% (6)**	**18.5% (12)**
Depression	0% (0)	0% (0)	**3.7% (4)**	**15.2% (7)**	**2.4% (4)**	**10.8% (7)**
Somatic	0% (0)	0% (0)	3.7% (4)	2.2% (1)	2.4% (4)	1.5% (1)

*Significant differences obtained in Fisher Exact Test are presented in bold.*

### Discussion

In Experiment 1, we investigated the role of the educational stage (primary versus secondary school) in exacerbating the internalizing symptomatology in DD as compared to controls. We found higher levels of internalizing symptoms in secondary compared to primary school both in DD and in controls. This result is in line with numerous studies that described that the transition from primary to secondary school, which coincides with the beginning of adolescence, represents a particularly difficult period. This period requires the children to face a series of physical, social and relational challenges both in the school and in the family and in the social sphere which could create fertile ground for the emergence of emotional suffering. In this context, when learning difficulties are added to the adolescence challenges, the risk of developing anxious, depressive and somatic symptoms seems to increase significantly. Indeed, our findings proved that not only the DDs shown higher internalizing symptomatology levels than controls, but internalizing symptoms tend to worsen in secondary school more in DDs than in controls. The relationship between learning problems and internalizing symptoms in children and adolescents is not surprising given the primacy of school experiences in shaping the social, emotional, and mental health functioning of young people (Waters et al., 2010). Particularly relevant is the result of anxiety dimension. In the DD group, the anxiety levels resulted significantly higher in secondary than in primary school, whereas in the control group, the symptomatology is lower in secondary school. Anxiety, in particular, school-anxiety, has been found typically linked to the presence of specific learning disorders (Geisthardt and Munsch, 1996; Wenz-Gross and Siperstein, 1998; Goldston et al., 2007). According to the secondary reaction theory, anxiety develops as a result of learning difficulties (Nelson and Harwood, 2011): children, at an early age, learn the importance placed by academic success by their parents and teachers. Therefore, children who struggle to learn and master academic skills can develop an anxiety reaction in anticipation of a possible academic failure combined with the frustration of not meeting the expectations of their parents and teachers (Scott, 2003). At the same time, the feeling of anxiety can be the biggest obstacle to learning, especially for DD, preventing the adequate degree of concentration and clarity required by the study and contributing to avoidance academic work mechanisms (Cohen, 1986). In this context, anxiety can establish a vicious circle that continues to feed itself, creating increasingly important academic and emotional consequences. As our results highlighted, the percentage of DD cases with clinically relevant scores increases significantly in secondary school (especially as regard anxiety symptoms). Moreover, this result confirms that the presence of dyslexia, as claimed by numerous authors in the literature, is configured as a risk factor for emotional symptoms. It is therefore important to investigate the possible risk factors that explain the link between learning difficulties and worsening internalizing symptoms (Klassen et al., 2011).

## Experiment 2

Following the main finding of Experiment 1 we investigated the role of risk factor for the deepening of internalizing symptomatology. However, as reported in the literature, specific learning difficulties may not be the only factor contributing to the development of internalizing problems in DD children. According to developmental health theories, environmental factors such as academic stressors associated with schooling interact with biological predispositions to negative affectivity, resulting in anxiety and depression in children and adults (Zahn-Waxler et al., 2000). Moreover, the presence of specific learning problems, contribute to creating a repeated academic failure cycle results in a feeling of frustration, inferiority and low self-efficacy (Klassen et al., 2011). Repetitions of emotionally stressful and maladaptive situations can contribute to the creation of psychological factors (i.e. self-esteem, perceived quality of life, social support and decision-making strategies) that could have an impact on internalizing symptoms in DD during adolescence.

On the basis of these considerations, further studies are required to investigate the risk factors for internalizing symptoms in adolescent populations with DD, paying particular attention to contextual factors that are associated with transitioning from primary to secondary school. Without specifically referring to the adolescent population, extant studies have found that internalizing symptoms associated with DD were generally related to psychological discomfort, low self-esteem (LaGreca and Stone, 1990; Riddick et al., 1999; Humphrey and Mullins, 2002; Terras et al., 2009), emotional and behavioral difficulties (Snowling et al., 2007), as well as lower academic self-esteem (Zeleke, 2004) and a lack of motivation (Rheinberg, 2006). Furthermore, some studies showed that maladaptive coping strategies often characterize DD responses to learning difficulties (Firth et al., 2013) and constitute potential risk factors for internalizing symptoms (Alexander-Passe, 2006). In the field of problem-solving conceptualization, the decision-making styles were considered as an expression of individual coping strategies (Çolakkadıoğlu and Deniz, 2015). In addition, recent literature has shown that internalizing symptoms could be related to low cognitive ability (Flouri et al., 2018).

Experiment 2 specifically focused on the adolescent population. More precisely, we investigated the role of specific contextual and subjective factors that, in interaction with DD, contribute to the increase or decrease of symptom severity. To the best of our knowledge, no previous study has examined the association between anxiety, depression and somatic symptoms with the contextual and subjective factors investigated in the present study (i.e. decision-making factors, general cognitive ability, and perceived quality of students’ life). We expected to find that the internalizing symptoms in DD are exacerbated by low self-esteem and poor social relationships, whereas individual cognitive factors such as motivation and general intelligence were expected to be protective factors.

### Methods

#### Participants

Forty-four students with dyslexia (2 females, 42 males) and 51 age-matched controls (12 females, 39 males) attending an upper secondary technical school “IPSIA Comandini” participated in the study (mean age 14.9 years; SD = 0.89). Informed consent was appropriately obtained from the parents of all participants.

As fully described in experiment 1, the inclusion and exclusion criteria adopted were those recommended by Consensus Conference on Specific Learning Disorders promoted by the Italian National Institute of Health (Lorusso et al., 2014) for diagnosis of developmental dyslexia. Informed consent was appropriately obtained from the parents of all participants. Descriptive statistics of the sample of experiment 2 are reported in Table 4.

**TABLE 4:** Descriptive statistics of general intelligence (IQ) and reading test parameters (*Z* scores) in the experiment 2.

Group	*C*	DD
	Mean (SD)	Mean (SD)
PM Raven’S IQ	107.45 (11.42)	104.44 (12.53)
Time in word reading	0.10 (0.85)	3.73 (4.53)
Errors in word reading	−0.20(0.80)	4.51 (4.04)
Time in non-word reading	−0.41(0.86)	1.83 (2.78)
Errors non-word reading	−0.62(0.54)	1.07 (1.68)
Comprehenshion Test	4.93 (2.00)	4.78 (2.16)

*Mean and standard deviation (SD) are represented in Control (C) and Developmental Dyslexia (DD) group.*

#### Instruments

The reading and cognitive tests were the same as those described in Experiment 1.

Internalizing symptomatology was assessed by the Italian version of the Child Behavior Checklist, Youth Self Report version (CBCL_YSR/11-18 – Achenbach et al., 2001). The CBCL_YSR is a standardized questionnaire for the identification of emotional/behavioral problems and social competencies in children and adolescents. The CBCL_YSR is composed of 118 items. The subject is required to score each item according to a three-point Likert scale (0: not true; 1: somewhat or sometimes true; 2: very true or often true). Eight symptom scales were calculated as a sum of a group of specific items: Anxious/Depressed scale, Withdrawn/Depressed scale, Somatic Complaints scale, Social Problems scale, Thought Problems scale, Attention Problems scale, Rule-Breaking Behavior scale, and Aggressive Behavior scale. The internalizing symptomatology was evaluated considering the Anxious/Depressed scale (evaluating fear of school, fearfulness, crying, perfectionism, guilt, general worries, suicidal thoughts and/or attempted suicide), Withdrawn/Depressed scale (which measures loneliness, shyness, sadness, lack of energy, withdrawnness), Somatic Complaints scale (accounting for problems with sleep and different somatic symptoms). Moreover, the Total scale for Competence (as a sum of a scale concerning sport, employment and hobby activities, school competence scale and social competence scale) was applied as a measure of general social and school functioning. For all the scales, raw scores were transformed into T-scores according to standardized scores. Scores above 60 were used to indicate deviant behaviors as compared to normative scores for age and gender.

The Italian version of the Melbourne Decision Making Questionnaire (MDMQ; Nota and Soresi, 2000) was used to identify the subject’s decision-making style. Four decision-making styles are considered: 1. Avoidance of problems; 2. Procrastination in dealing with problems; 3. Vigilance, i.e. being alert to problems; and 4. Hypervigilance, namely an excessive sense of alertness toward school situations perceived as problematic.

The student’s quality of life was investigated using the Clipper test, an Italian validated test measuring the student’s life satisfaction (Soresi and Nota, 2003). Seven subscales were considered: 1. General satisfaction related to school experience; 2. Sense of autonomy, i.e. the sense of self-sufficiency in school activities; 3. Relationships with peers or satisfaction with peer support; 4. Current satisfaction in school experience; 5. Satisfaction in relationships with family and family support; 6. Perceived recognition of self-efficacy, mainly related to school activities; and 7. Perceived social support.

#### Procedure

The students were individually assessed by an expert psychologist in two different sessions. In the first session, reading and cognitive performances were measured, while in the second session internalizing symptoms (CBCL_YRS), Decision Making style (MDMQ) and the Student’s Quality of Life (Clipper) were evaluated.

### Statistical Analysis

Four Generalized Linear Models were separately applied to evaluate the differences between DD and C groups on the different subscales calculated by the YSR version of CBCL self-report questionnaire: (1) Anxiety/Depression scale, (2) Depression/Withdraw, (3) Somatic symptoms, and (4) General competencies. The effect of Decision-Making factors (1. Avoidance of problems; 2. Procrastination in dealing with problems; 3. Vigilance; 4. Hypervigilance), Quality of life factors (1. General satisfaction related to school experience; 2. Sense of autonomy; 3. Relationships with peers; 4. Current satisfaction in school experience; 5. Satisfaction in relationships with family; 6. Perceived recognition of self-efficacy; and 7. Perceived social support) and general intelligence (Raven test scores) were taken into account. In each model, the different CBCL_YSR subscales were used as dependent variables, the Group (DD vs. C) was used as a subject factor, while Decision-Making factors, the Student’s Quality of Life factors and the general intelligence factor were the covariates. The Generalized Linear Model assumes that the dependent variable is linearly related to the factors and covariates via a specified link function. Moreover, the model allows for the dependent variable to have a non-normal distribution. The statistical assumption of the generalized linear model (statistical independence of observations, correct specification of link function) have been carefully checked and criteria were met.

### Results

All four Generalized Linear Models returned significant levels (Anxiety: Likelihood Ratio Chi-Square = 43.6, *p* = 0.01; Depression: Likelihood Ratio Chi-Square = 55.7, *p* < 0.001; Somatic: Likelihood Ratio Chi-Square = 47.7, *p* < 0.001; Competencies: Likelihood Ratio Chi-Square = 51.6, *p* < 0.001), attesting to the fact that the factors considered were, when taken together, predictive for internalizing symptomatology.

The effect of dyslexia *per se* was not predictive for increased anxiety, depressive or somatic symptoms (see Table 5). Only when considering the presence of dyslexia in interaction with the tendency for hypervigilance, higher level of internalizing symptoms were found in DD as compared to controls [Anxiety/Depression: b(DD) = 43.2; b(C) = 35.9; Depression/Withdraw: b(DD) = 45.7; b(C) = 27.5; Somatic symptoms b(DD) = 51.3; b(C) = 38.7]. Depressive symptoms were higher in DD as compared to C when associated with the perception of negative relationships with peers [b(DD) = 45.0; b(C) = 27.1]. Somatic symptoms resulted higher in DD when linked with a low level of self-efficacy [Somatic b(DD) = 50.9; b(C) = 38.1].

**TABLE 5:** Effect of Dyslexia and interaction between dyslexia and Quality of life (QL) and Decision making (DM) and general cognitive factors on internalizing symptoms and competences evaluated by means of Generalized Linear Models.

	Dyslexia	Dyslexia by DM1	Dyslexia by DM2	Dyslexia by DM3	Dyslexia by DM4	Dyslexia by QL1	Dyslexia by QL2	Dyslexia by QL3	Dyslexia by QL4	Dyslexia by QL5	Dyslexia by QL6	Dyslexia by QL7	Dyslexia by GI
Anxiety/Depression	0.12	0.88	1.46	2.98	9.17*	0.81	0.96	4.83	2.93	0.33	1.54	0.90	1.48
Depression/Withdrow	0.67	1.40	0.70	2.01	13.60**	3.75	3.69	8.94**	1.24	3.01	4.30	3.17	2.09
Somatic symptoms	0.50	0.85	2.05	1.28	14.01**	2.19	0.98	3.27	2.30	3.42	5.97*	1.63	2.05
Competence	0.01	0.91	0.39	5.73*	0.19	6.39*	4.62	6.00*	1.06	0.32	1.33	2.03	4.89

DM1: Decision making 1 – Avoidance; DM2: Decision making 2 – Procrastination; DM3: Decision making 3 – Vigilance; DM4: Decision making 4 – Hypervigilance. QL1: Quality of life 1 – General satisfaction; QL2: Quality of life 2 – Autonomy; QL3: Quality of life 3 – Relationship with peers; QL4: Quality of life 4 – Current satisfaction; QL5: Quality of life 5 – Satisfaction for family relationships; QL6: Quality of life 6 – Perceived recognition of self-efficacy; QL7: Quality of life 7 – Perceived social support; GI: General Intelligence. *p < 0.05. **p < 0.01.

Analysis regarding the Competence scale confirmed that the DD and C groups did not differ in competences but that DD subjects were more strongly affected by risk and protective factors than those in C. In DD general satisfaction increased the sense of competencies more than in C [b(DD) = 8.7; b(C) = 7.1]. Negative relationships with peers [b(DD) = 9.0 b(C) = 6.9], affected DD competency levels more than for the controls. The effect of vigilance in interaction with dyslexia was almost significant (*p* = 0.057), showing a higher effect of vigilance in DD’s sense of competencies as compared to the control [b(DD) = 8.6; b(C) = 6.9].

No effect of general intelligence in interaction with dyslexia was found for any of the scales (see Table 5).

### Summary of Results of Experiment 1 and 2

The results can be summarized as follow:

In Experiment 1, we found higher levels of anxiety, depressive and somatic symptoms in the DD group compared to the controls. We found differences regarding the context of assessment: higher scores were obtained in the clinical setting compared to the screening setting.

The differences concerning anxiety were related to the dissimilarities between DD and C in the transition from the primary to the secondary school. In the DD group, anxiety levels were higher in the secondary rather than in primary school, whereas in the C group the anxious symptoms were lower in the secondary school. Higher levels of depressive and somatic symptoms in the secondary school were found both in DD and C groups; the DD group featured a greater increase compared to C, but the difference was not statistically significant.

Analysis of the frequencies of the clinically relevant score (i.e. the number of subjects whose score was above two standard deviations of the normative data) generated important findings. In the primary school, no DD featured clinical levels of depression symptoms and 5.3% had clinical levels of anxiety symptoms. In the secondary school, 23.9% of DD cases manifested clinical levels of anxiety symptoms and 15.2% had clinical levels of depression symptoms. Therefore, the transition from primary to secondary school in the DD cohort resulted in a percentage increase of 18.6% in cases of anxiety and 15.2% of depression.

In Experiment 2, the results on an upper secondary school cohort confirmed higher levels of anxiety, depression and somatic symptoms in DD compared to the C groups. However, the severity of internalizing symptoms did not depend on the dyslexia *per se*, but was instead related to dyslexia in connection with hypervigilance, lack of good peer relationships and low level of self-efficacy and self-esteem.

## General Discussion

The present study was designed to shed light on the role of contextual risk factors on increased internalizing symptoms in DD. The results add to the literature by finding that contextual and individual characteristics are important risks factors which worsen internalizing symptoms in DD. Specifically, while the results from the first experiment showed that internalizing symptoms associated to DD occur more frequently and are more severely during adolescence, those from the second experiment demonstrated that the worsening of the symptoms manifested in adolescence can be explained by a lack of sound peer relationships, low self-efficacy, low self-esteem and hypervigilance in school-settings.

Specifically, among all the internalizing symptoms considered in the present study, school anxiety in DD proved to be significantly higher in secondary school compared to primary school, while symptoms decreased in C. School anxiety is strictly connected with worries about dysfunctional contextual situations and relationships with others that occur in adolescence. It is not surprising that ongoing academic struggles lead to increased anxiety in academic settings. This, in turn, breeds generalized anxiety due to the frustration of not meeting one’s own and parents’ expectations (Scott, 2003). These findings can be better explained in the light of the results obtained in the second experiment.

Referring to a “conundrum of failure,” Tanner (2009) sought to highlight the importance of factors that impact on the self-perception and self-efficacy of DD from the first experience of school, through adolescence till adulthood. From this point of view, adolescence can be considered a key period in which the experienced school difficulties associated to DD could be resolved through adequate coping strategies or could be exacerbated by maladaptive coping strategies leading to internalizing symptoms (Firth et al., 2013). A well-designed study by Alexander-Passe (2006), describing the maladaptive and effective coping strategies in DD concerning academic self-esteem and depression, demonstrated the importance of considering these strategies as risk factors for negative consequences of DD.

In line with the above cited studies, the second experiment confirmed that in DD the significantly higher levels of anxiety symptoms in secondary school are related to a generalized worries, low self-esteem, low self-efficacy and hyperactivation in facing problems. These maladaptive coping strategies could, in turn, be exacerbated by higher academic demands coupled with the need to develop new ways of relating to others. In fact, whilst academic settings are more structured and more evaluation/outcome-focused compared to primary school settings, adolescents need to develop more flexible social skills in order to adapt to new types of relationships with peers and adults. The contrasting need for structure, on the one hand, flexibility on the other and the maladaptive coping strategies mentioned above might create a state of anxiety which arguably fuel a vicious cycle. The DD adolescents showed higher internalizing scores compared with C adolescents, but it is important to acknowledge that the presence of DD *per se* does not increase the risk of clinical psychopathology. In this respect, our results showed that peer relationships could be considered as a protective factor contributing to emotional wellbeing, despite learning disability.

The reported results should also be interpreted in light of the limitations of our study. A first limitation concerns the sample characteristics in Experiment 2, i.e. the school was a technical institute in which the vast majority of students are males. Thus, the results from the second experiment should be interpreted as referring to the male DD population. In light of the findings reported by Alexander-Passe (2006), who demonstrated the effect of the differences of male and female DD coping strategies, further studies are needed to investigate whether the results from the present study could be generalized to the female adolescent DD population. Another limitation concerns the age range of the sample, which is limited to early adolescence. Subsequent research should investigate older children in order to further assess the development of symptomatology.

In conclusion, our findings indicate that DD internalizing symptoms associated with hypervigilance, low self- esteem and low self-efficacy in adolescents with DD should be promptly recognized and treated. There is an urgent need for the development of effective interventions to address emotional wellbeing and to reduce the development of maladaptive coping strategies and distorted self-perceptions. Addressing this would arguably prevent the emergence of severe internalizing symptoms (McNulty, 2003; Singer, 2007; Westwood, 2008; Firth et al., 2013). Furthermore, as suggested by our results, particular attention should be paid to those protective factors that could prevent the development of severe anxious, depressive and somatic symptoms. Therefore, interventions should include activities and strategies aimed at increasing the motivation and self-esteem of adolescents with dyslexia and facilitating the construction of effective peer relationships. Further longitudinal studies are also needed to find the most effective screening tools for detecting early signs of internalizing symptoms and associated risk/protective factors.

## Data Availability Statement

The datasets generated for this study are available on request to the corresponding author.

## Ethics Statement

The reported work has been carried out in accordance with the Code of Ethics of the World Medical Association (Declaration of Helsinki). The studies involving human participants were reviewed and approved by the Comitato di Bioetica, Università di Bologna. Written informed consent to participate in this study was provided by the participants’ legal guardian/next of kin.

## Author Contributions

SG, MB, LG, ET, and DF designed the experiments. SG, MB, LM, and SM wrote the manuscript. SG, LM, and SM collected the data. SG, MB, and LM performed the data analysis. All authors read and reviewed the manuscript.

## Conflict of Interest

The authors declare that the research was conducted in the absence of any commercial or financial relationships that could be construed as a potential conflict of interest.
