# Supplementary material for: Internalizing Symptoms in Developmental Dyslexia: A Comparison Between Primary and Secondary School
Source: Front Psychol. 2020 Mar 24;11:461. doi: 10.3389/fpsyg.2020.00461 (PMC7105858; doi:10.3389/fpsyg.2020.00461)
Supplement: Supplementary file 1 [file Table_1.DOCX]

**Appendix 1**

**Differences between DD and C on Anxiety, Depression and Somatic Symptoms SAFA subscales**

The MANOVA applied to the Anxiety subscales showed significant differences between the DD and C groups for Social Anxiety (F(1, 226)=4.08; p=.04; partial-η2=.02), and School Anxiety (F(1, 226)=7.32; p<.01; partial-η2=.03). No significant differences were found for Separation Anxiety (F(1, 226)=.45; p=.51; partial-η^2^<.01) nor for Generalized Anxiety (F(1, 226)=2.55; p=.11; partial-η2=.01). The estimated mean scores of all the anxiety symptoms subscales were higher in the DD group than in the C group.

A significant interaction group by school effect was obtained for School Anxiety (F(1, 226)=4.82; p=.02; partial-η2=.02) subscale, but not for the Social Anxiety (F(1, 226)=3.00; p=.09; partial-η^2^=.01), Separation Anxiety (F(1, 226)=.08; p=.13; partial-η2=.01) and Generalized Anxiety (F(1, 226)=2.84; p=.09; partial-η2=.01) subscales. DD showed higher level of school anxiety when attending secondary school as compared to C children.

From the analysis of clinically relevant scores, the DD group showed a significantly higher percentage of cases with Generalized Anxiety clinical scores in Secondary school (Fisher's exact test; p=.02) compared to C group but not in Primary school (Fisher’s exact test; p=1.00). Social Anxiety and School anxiety symptoms scale showed the same results: significant differences between DD and C children were obtained in Secondary school (Fisher’s exact test; Social p=.001; School p=.02) but not in Primary school (Fisher's exact test; Social p=1.00; School=0.44). Separation Anxiety symptoms subscale did not show any differences in Primary and Secondary school.

**Differences between DD and C on depressive symptoms subscales**

From the MANOVA, the univariate analysis of variance showed significant differences between the DD and C groups for Irritable Mood (F(1, 226)=5.62; p=.02; partial-η2=.02), Inadequacy (F(1, 226)=9.98; p=.002; partial-η2=.04), Insecurity (F(1, 226)=12.22; p=.001; partial-η2=.05). No significant differences were found for General Depression (F(1, 226)=2.07; p=.15; partial-η^2^<.01), Anhedonia (F(1, 226)=.88; p=.77; partial-η2<.01) and sense of Guilt (F(1, 226)=.02; p=.88; partial-η2<.01) and Despair (F(1, 226)=.14; p=.71; partial-η2<.01). The estimated mean scores of all the depression subscales were higher in the DD group than in the C group.

No significant interaction effect group by school was found, however the estimated means showed higher level of symptomatology in Secondary school in DD children compared to C children in all depression subscales.

From the analysis of clinically relevant scores, DD group showed a significantly higher percentage of cases with sense of Guilt clinical scores in Secondary school (Fisher's exact test; p=.02) but not in primary school (Fisher’s exact test; p=1.0) as compared to C. Irritable mood, Inadequacy and Insecurity subscale showed the same results: significant differences between DD and C children were obtained in Secondary (Irritable mood: Fisher’s exact test; p=.007: Inadequacy: Fisher’s exact test; p=.007; Insecurity: Fisher’s exact test; p=.009) but not in Primary school (Irritable mood: Fisher’s exact test; p=.42: Inadequacy: Fisher’s exact test; p=.14; Insecurity: Fisher’s exact test; p=.054). No significant differences between DD and C children in Primary (Fisher’s exact test; Depressed mood: p=.24; Despair: p=.13 ), nor in Secondary (Fisher’s exact test; Depressed mood: p=.17; Despair: p=.12) school were found for Depressed Mood and Despair subscale.

**Differences between DD and C on Somatic Symptoms and Hypochondria subscales**

From the MANOVA, no significant differences were found between C and DD group in Somatic Symptoms (F(1, 226)=2.66; p=.10; partial-η2=.01), nor for Hypochondria (F(1, 226)=.57; p=.45; partial-η2=.002). No interaction group by school effect was found (Somatic Symptoms: F(1, 226)=.69; p=.41; partial-η2=.003); Hypochondria: F(1, 226)=2.09; p=.15; partial-η2=.009). Estimated mean score of the Somatic Symptoms subscale were higher in DD group than in C group.

The analysis of somatic symptoms subscales clinically relevant scores did not find any differences between C and DD groups in the percentage of cases with clinical scores in primary (Fisher’s exact test; Somatic Symptoms: p=1.0; Hypochondria: p=.57 ) and secondary school (Fisher’s exact test; Somatic Symptoms p=1.0; Hypochondria: p=1.0).
